# Condom use in combination with ART can reduce HIV incidence and mortality of PLWHA among MSM: a study from Beijing, China

**DOI:** 10.1186/s12879-018-3026-8

**Published:** 2018-03-13

**Authors:** Lili Tao, Min Liu, Shuming Li, Jue Liu, Ning Wang

**Affiliations:** 1 Beijing Chaoyang District Centre for Disease Control and Prevention, No. 25 Panjiayuan Huaweili, Chaoyang District, Beijing, China; 2 Peking University, School of Public Health, No. 38 Xueyuan Road, Haidian District, Beijing, China; 3 National Center for AIDS/STD Control & Prevention, No. 155 Chang Bai Road, Changping District, Beijing, China

**Keywords:** Condom use, Antiretroviral therapy (ART), Human immunodeficiency virus (HIV)

## Abstract

**Background:**

Condom use and antiretroviral therapy (ART) are effective means to prevent and control HIV transmission. We aimed to assess the effect of condom use in combination with antiretroviral therapy (ART) on HIV incidence and mortality among men who had sex with men (MSM) in Beijing, China.

**Methods:**

We evaluated the effect of condom use, ART, and the combination of both among people living with HIV/AIDS (PLWHA) of MSM in the Chaoyang District of Beijing using the Asian Epidemic Model (AEM). Evaluation indicators included absolute risk reduction (ARR) and the percentage of relative risk reduction (RRR %).

**Results:**

HIV incidence and prevalence declined substantially when condoms were used by MSM in Chaoyang from 2003 to 2013. The ARR of HIV incidence was from 0 to 0.91% and the RRR% was from 0 to 43.93%. The ARR of HIV prevalence was from 0 to 3.79% and the RRR% was from 0 to 31.53%. The HIV mortality rate decreased substantially (ARR from 0 to 1.75%, and RRR% from 0 to 40.03%) when ART was implemented. When condom use combined with ART was implemented in MSM in 2003–2013, HIV incidence declined substantially (ARR from 0 to 0.99%, and RRR% from 0 to 46.11%). HIV prevalence was also reduced with an ARR from 0 to 3.5%, and an RRR% from 0 to 29.88%. The HIV mortality also declined substantially (ARR from − 0.01% to 1.02%, RRR% from − 1.44% to 39.98%).

**Conclusions:**

Among MSM, a combination of condom use and ART reduces both HIV incidence and mortality caused by HIV. Combining these methods results in a more effective prevention and control of HIV.

## Background

High-risk behaviour intervention is one of the most effective means to prevent and control HIV transmission [1]. Condom use is an effective method to prevent the sexual transmission of HIV, the effect of which has been clearly demonstrated in China [2]. Antiretroviral therapy (ART) can effectively decrease the viral load in the blood and body fluids of people living with HIV/AIDS (PLWHA), thereby reducing HIV transmission [3, 4] and mortality. Currently, Pre-Exposure Prophylaxis (PrEP) has been gradually implemented as a preventative measure in populations at high risk of HIV infection [5]. ART was first introduced in 2003 in Chaoyang District, Beijing. With the implementation of the national policy “Four Frees and One Care” and the adjustment of the initial eligibility threshold for therapy, [6] ART coverage increased yearly, with larger numbers of PLWHA benefiting from ART. ART has been shown to prolong the survival of PLWHA and to improve the quality of life [7].

Chaoyang District, located in the northeast of Beijing, covers an area of 470.8 km^2^. Notably, Chaoyang is the largest urban district of Beijing with a population of 5,050,000, which is approximately one-fourth of the capital’s total population. The first case of HIV infection in Chaoyang was reported in 1990. By December 31, 2014, a total of 5481 PLWHA cases had been reported in Chaoyang, with male homosexual transmission accounting for 71.2% of all cases [8]. HIV-positive men who had sex with men (MSM) comprised the largest contingent of HIV infections. In 2003, Chaoyang District implemented the National AIDS Comprehensive Prevention and Control Demonstration Pilot Project. The project focused on peer education, condom use among MSM, and ART among PLWHA. As the project progressed, the rates of condom use and ART coverage improved among MSM in the area.

We used the Asian Epidemic Model (AEM) [9] to evaluate condom use, ART, and the combined effect of both methods on PLWHA who were MSM. The AEM was primarily developed to estimate epidemics and to simulate the spread of HIV in Asian countries [9–11]. With this model, we used the concept of “a Cause-Deleted Life Table” in a fixed area [12] and a relatively stable population to estimate the effect of the above methods on HIV incidence and HIV-related deaths of PLWHA.

## Methods

### Aim

We aimed to assess the effect of condom use in combination with antiretroviral therapy (ART) on HIV incidence and mortality from 2003 to 2013 among MSM in Beijing.

### Study design

#### The Asian epidemic model

The model we used to evaluate the effectiveness of interventions was AEM (version 4.0), which was developed by Dr. Tim Brown et al.*,* with support from the United States Agency for International Development (USAID). AEM is a full process model that is used to estimate and project the spread of HIV and to assess interventions in Asian areas [13]. The model calculates the numbers of new HIV infections in each subgroup based on different routes of transmission. It can also be used to calculate the number of current HIV infections and HIV-related deaths [14].

1. Calculation of the number of new HIV infections.

The number of new HIV infections (new HIV cases in one year) was calculated based on sexual frequency, multiplying the probability of HIV infections per dangerous behaviour and corrections for co-infection with HIV transmission.

For example, the number of new sexual infections of clients by direct sex workers was calculated as previously described [10]:

$$ \mathrm{n}={\mathrm{p}}_{{\mathrm{f}}_{\mathrm{m}}}{\mathrm{X}}_1{\mathrm{V}}_{1\mathrm{a}}(t)\left(1-{\mathrm{C}}_{1\mathrm{a}}(t)\right)\left(\frac{{\mathrm{Y}}_{3\mathrm{a}}}{{\mathrm{X}}_{3a}+{\mathrm{Y}}_{3\mathrm{a}}}\right)\mathrm{x}\left[{\mathrm{C}}_{\mathrm{std}\_\mathrm{m}}{\mathrm{F}}_{\mathrm{std}\mathrm{a}(t)}+\left(1-{\mathrm{F}}_{\mathrm{std}\mathrm{a}}(t)\right)\right]+\left[{\mathrm{C}}_{\mathrm{cc}}{\mathrm{F}}_{\mathrm{cc}}(t)+\left(1-{\mathrm{F}}_{\mathrm{cc}}(t)\right)\right] $$

X_1_ was the number of uninfected clients, V_1a_(*t*) was the frequency of contact with sex workers, C_1a_(*t*) was the level of condom use, and X_3a_ and Y_3a_ were the number of uninfected and infected direct sex workers. The additional components [C_std_m_F_stda(*t*)_ + (1 − F_stda_(*t*))] and [C_cc_F_cc_(*t*) + (1 − F_cc_(*t*))] at the end of the expression represented the adjustments to the transmission for STD enhancement of transmission and the influence of circumcision, respectively.

The number of new HIV infections in each subgroup by different routes of transmission was calculated in the same way.

(2) Calculation of the number of current HIV infections (people living with HIV/AIDS), AIDS patients, and HIV-related deaths.

After the number of new HIV infections was calculated in each subgroup for each year, we could determine the numbers of new AIDS- and HIV-related deaths based on the progression of an HIV infection developing into AIDS and resulting in death. We obtained the number of current HIV infections using the cumulative HIV infections minus the sum of new AIDS- and HIV-related deaths.

We obtained the number of new HIV infections, current HIV infections, and HIV-related deaths among MSM during 2003–2013 from the results of the general population in Chaoyang District. We then changed the input on behavioural and ART parameters and ran the AEM program again. Next, we determined annual HIV infections and HIV-related deaths under different levels of ART and condom use. The difference between these two results was taken to represent the effect of behaviour and treatment intervention [9].

### Source of data [15]

We divided the population into the following four subgroups according actual transmission route in Chaoyang: (1) female sex workers (FSW) and clients through heterosexual behaviour; (2) MSM including male sex workers (MSW) through homosexual behaviour; (3) IV drug users (IDU) by sharing syringes; and (4) the general population.

1. Population size: Demographic data were derived from the Statistical Yearbooks (1990 to 2010) of the Chaoyang District of Beijing, China [16, 17].
2. Behavioural parameters: Data related to the trends in behavioural changes, including condom use (2003 to 2013) (Table 1), proportions of needle sharing among IDUs (1999 to 2012), and sexual behaviour among IDUs and sex workers (1999 to 2010), were primarily derived from sentinel surveillance programmes offered by the Beijing Chaoyang District Center for Disease Control and Prevention [8, 18–22].
3. HIV prevalence: Data on HIV prevalence was collected from the relevant population groups including FSW, IDU, and MSM at the sentinel surveillance points (2003 to 2013) in the Chaoyang District of Beijing [23–26] (as shown in Table 1).
4. ART-related parameters: ART coverage data (2003–2013) was obtained from the Report of Beijing Chaoyang District Health Bureau, and other ART-related parameters were obtained from the Beijing Chaoyang District AIDS Comprehensive Prevention Information System in China (2003 to 2013) and other published references (Table 1) [27, 28].
5. Epidemiological parameters: Probabilities of HIV transmission via different routes, including vaginal intercourse transmission from males to females, vaginal intercourse transmission from females to males, transmission from anally insertive partners to receptive partners, transmission from anally receptive partners to insertive partners, and shared needles for intravenous drug injections, were from published references [29–32].

**Table 1:** ART-related parameters, condom use and parameters of HIV prevalence among key affected population in Chaoyang District in 2003–2013

Year	ART coverage (%)	Initiation on CD4 cell count (/μl)	HIV prevalence of FSW (%)	HIV prevalence of male IDU (%)	HIV prevalence of female IDU (%)	HIV prevalence of MSM (%)	Condom use between MSM and MSM (%)	Condom use between MSM and MSW (%)
2003	5.06	200	0.26	8.01	7.83	1.34	42.00	55.00
2004	10.91	200	0.34	7.68	6.98	1.55	44.20	56.08
2005	13.81	200	0.50	6.82	8.00	3.23	46.10	62.10
2006	18.27	200	0.29	6.48	6.14	4.81	48.34	62.74
2007	21.36	200	0.71	6.04	5.66	4.50	50.08	63.30
2008	20.98	350	0.62	5.86	6.45	5.40	51.12	63.78
2009	20.11	350	0.07	6.22	6.06	6.04	51.30	64.10
2010	21.71	350	0.15	4.26	6.67	6.09	52.09	64.56
2011	24.14	350	0.20	3.65	3.00	5.60	53.48	65.24
2012	27.40	350	0.10	3.95	2.81	6.87	54.48	66.08
2013	29.72	350	0.10	3.70	2.85	7.12	56.00	66.70

*ART* Antiretroviral therapy, *FSW* Female sex workers, *HIV* Human immunodeficiency virus, *IDU* IV drug user, *MSM* Men who have sex with men, *MSW* Male sex worker

Source: ART coverage, [33] initiation on CD4 cell count, [34] HIV prevalence of FSW, IDU (male and female) and MSM, condom use between MSM and MSM, condom use between MSM and MSW were from the surveillance points in Chaoyang District of Beijing in China (2003–2013) and research findings [23–26]

### Setting of the study

#### Research hypothesis

We used AEM to estimate the numbers of new HIV infections, current HIV infections, and HIV-related deaths among MSM in Chaoyang. The estimated results were identified because of the combined intervention of AIDS in Chaoyang in this study.

We divided MSM into different scenarios based on the intervention measures: with comprehensive intervention, without condom use, without ART, and without a combination of both condom use and ART. We analysed the differences between new HIV infections, current HIV infections, and HIV-related deaths.

Determining the effect of comprehensive interventions: we assumed that the estimated numbers of new HIV infections, current HIV infections, and HIV-related deaths among MSM in 2003–2013 based on the AEM were the effect of comprehensive interventions.

Determining the effect of interventions other than condom use: we assumed that the rate of condom use per year remained at the level of 2003 (the rate of condom use per year between MSM and MSM was 42.00%; between MSM and MSW, the rate was 55.00%). Holding other factors unchanged, we calculated the numbers of new HIV infections, current HIV infections, and HIV-related deaths among MSM from 2003 to 2013, which we considered to be the effect of other interventions apart from condom use.

Determining the effect of interventions other than ART: we assumed that ART coverage and the CD4 threshold for treatment eligibility among PLWHA of MSM remained the same as the 2003 level (ART coverage was 5.06%; CD4 cell count ≤200 cells/μl). Holding other factors unchanged, we calculated the numbers of new HIV infections, current infections, and HIV-related deaths among MSM in 2003–2013, which we considered to be the effect of other interventions apart from ART.

Determining the effect of interventions other than combined condom use and ART: we assumed that ART coverage, the CD4 threshold for treatment eligibility among PLWHA of MSM, and the rate of condom use remained at the same level as 2003 (ART coverage was 5.06%; CD4 cell count ≤200 cells/μl; the rate of condom use between MSM and MSM was 42.00%; the rate of condom use between MSM and MSW was 55.00%). With other factors held unchanged, we calculated the numbers of new HIV infections, current HIV infections and HIV-related deaths among MSM in 2003–2013, which we considered to be the effect of other interventions apart from a combination of condom use and ART.

### Assessing index

In this study, we used absolute risk reduction (ARR) and relative risk reduction percentage (RRR%) to represent the effect of interventions. ARR is the absolute difference between rates of event incidence without and with an intervention. A larger ARR indicates a more effective intervention. The RRR% is a determination of the decrease in the relative risk of an adverse event (e.g., HIV infection) compared to the risk of an adverse event pre-intervention. Thus, the RRR% indicates a reduction in the degree of relative risk before and after an intervention.

The “events” in the following formula referred to new HIV infections, current HIV infections and HIV-related death.

Effect of condom use among MSM:

ARR = Event rates without condom use minus event rates with comprehensive interventions.

$$ RRR={\frac{\mathrm{Event}\ \mathrm{rates}\ \mathrm{with}\mathrm{out}\ \mathrm{condom}\ \mathrm{use}\hbox{-} \mathrm{event}\ \mathrm{rates}\ \mathrm{with}\ \mathrm{comprehensive}\ \mathrm{interventions}}{E\mathrm{vent}\ \mathrm{rates}\ \mathrm{with}\mathrm{out}\ \mathrm{condom}\ \mathrm{use}}}^{\ast }100\% $$

Effect of ART among PLWHA of MSM:

ARR = Event rates without ART minus event rates with comprehensive interventions.

$$ RRR={\frac{\ \mathrm{Event}\ \mathrm{rates}\ \mathrm{with}\mathrm{out}\ \mathrm{ART}\hbox{-} \mathrm{event}\ \mathrm{rates}\ \mathrm{with}\ \mathrm{comprehensive}\ \mathrm{interventions}}{\mathrm{Event}\ \mathrm{rates}\ \mathrm{with}\mathrm{out}\ \mathrm{ART}}}^{\ast }100\% $$

Effect of condoms in combination with ART among MSM:

ARR = Event rates without condoms in combination with ART minus event rates with comprehensive interventions.

$$ RRR={\frac{\mathrm{Event}\ \mathrm{rates}\ \mathrm{with}\mathrm{out}\kern0.5em \mathrm{condom}\ \mathrm{in}\ \mathrm{combination}\ \mathrm{with}\ \mathrm{ART}\hbox{-} \mathrm{event}\ \mathrm{rates}\ \mathrm{with}\ \mathrm{comprehensive}\ \mathrm{in}\mathrm{terventions}}{\mathrm{Event}\ \mathrm{rates}\ \mathrm{with}\mathrm{out}\ \mathrm{condom}\ \mathrm{in}\ \mathrm{combination}\ \mathrm{with}\ \mathrm{ART}}}^{\ast }100\% $$

### Modelling process

In this study, we used AEM to assess the impact of condom use in combination with ART on HIV incidence and mortality among MSM in Beijing.

First, we filled in many key inputs, including population size, behavioural parameters, HIV prevalence, ART-related parameters, and epidemiological parameters, which were shown in the “source of data.” After running the AEM, we gained much data about the number of new HIV infections, current HIV infections, and HIV-related deaths among MSM during 2003–2013 in the Chaoyang District of Beijing. This was a result of the status of the HIV epidemic with comprehensive interventions among MSM.

Next, we reset the inputs on the rate of condom use from 2003 to 2013 among MSM (remained at the level of 2003) in AEM and maintained the other inputs unchanged to allow for the analysis of the downstream impact of other interventions apart from condom use.

Then, we made an adjustment to the inputs on the ART coverage and the treatment eligibility from 2003 to 2013 (the same as the 2003 level) and held other inputs unchanged. We ran the AEM once again and obtained the effect of other interventions apart from ART.

Lastly, we readjusted the inputs both on the rate of condom use among MSM and on the ART coverage and the treatment eligibility from 2003 to 2013; the other factors remained unchanged. We obtained the effect of the other interventions apart from a combination of condom use and ART.

## Results

**Fig. 1:**
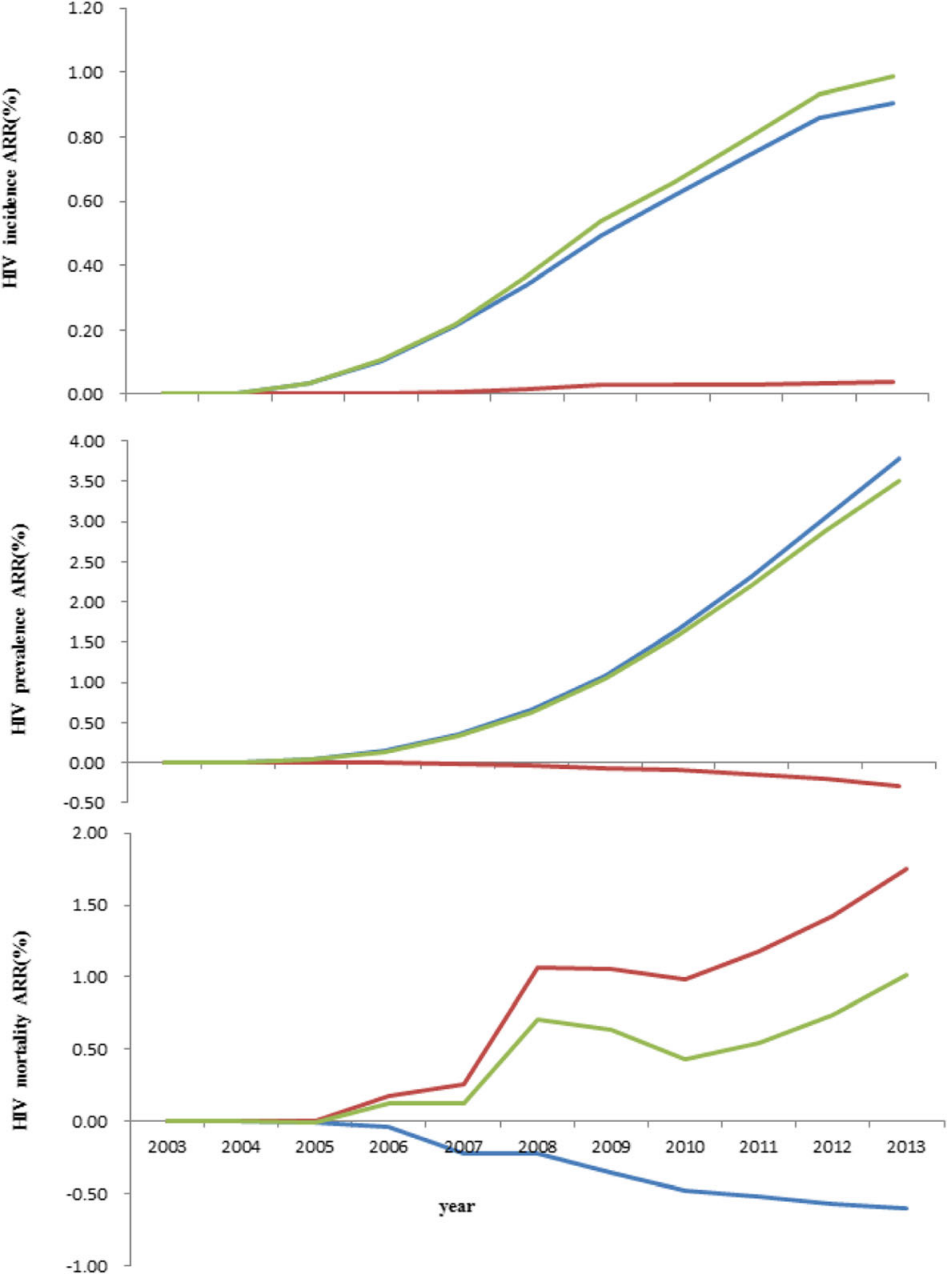
Impact of condom use, ART and combination of both on ARR of HIV infections among MSM and HIV mortality among PLWHA of MSM in 2003–2013.  Condom use, ART, ART combined with condom use

**Fig. 2:**
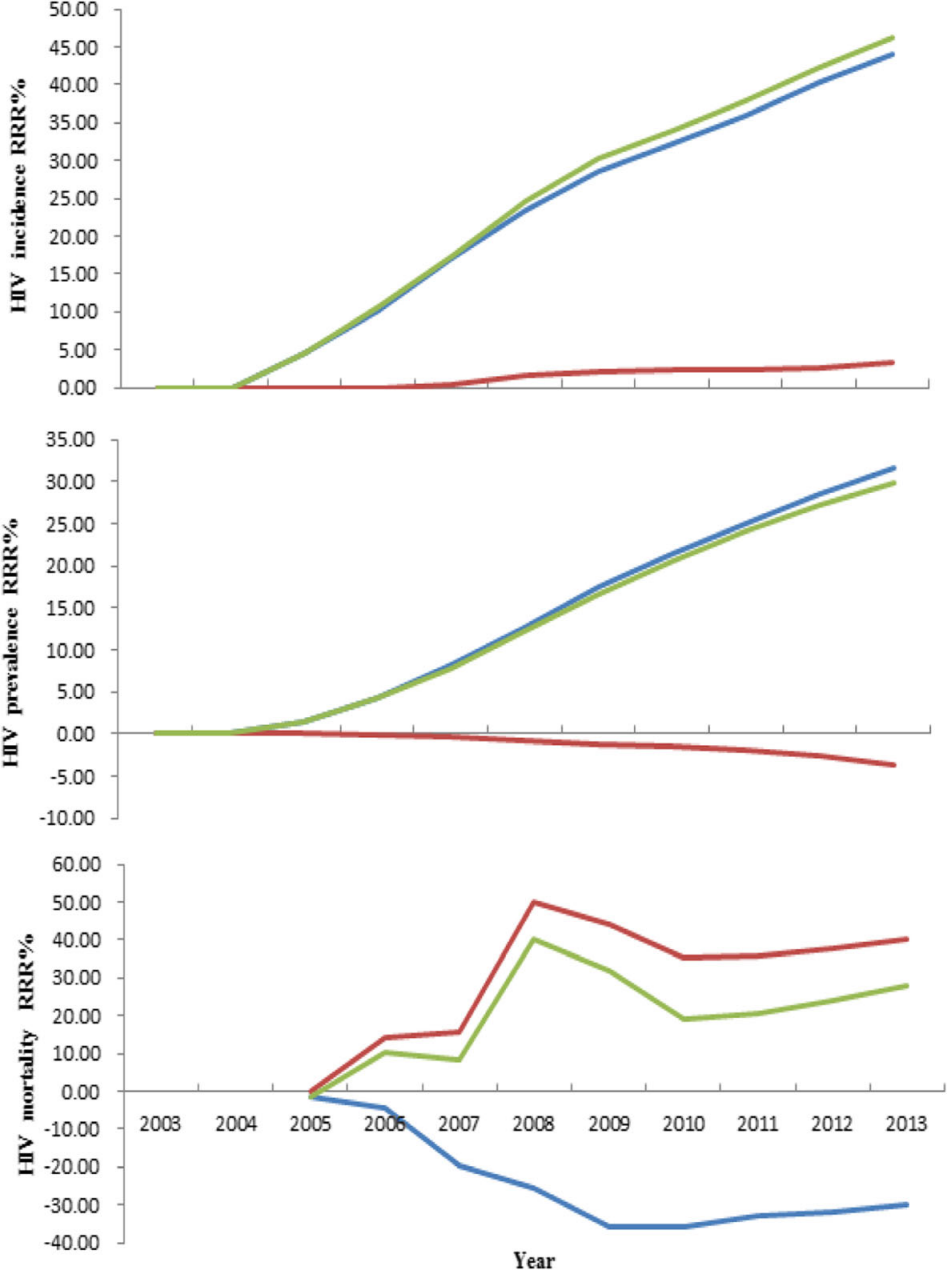
Impact of condom use, ART and combination of both on RRR% of HIV infections among MSM and HIV mortality among PLWHA of MSM in 2003–2013. Condom use, ART, ART combined with condom use

Through model fitting, we found that when condoms were used, HIV incidence decreased substantially among MSM between 2003 and 2013 in Chaoyang, with an ARR from 0 to 0.91%, and an RRR% from 0 to 43.93%. HIV prevalence also declined with an ARR of 0~ 3.79% and an RRR% of 0~ 31.53% between 2003 and 2013. HIV mortality showed a rising trend with ARR from -0.61% to 0% and RRR% from − 1.44% to 35.71% (Table 2, Figs. 1 and 2).

**Table 2:** Impact of condom use on ARR and RRR% of HIV infections among MSM and HIV mortality among PLWHA of MSM in 2003–2013

		HIV incidence (per year)				HIV prevalence (per year)				HIV mortality (per year)			
Year	Num	With comprehensive interventions (%/n)	Without condom use (%/n)	ARR (%)	RRR%	With comprehensive interventions (%/n)	Without Condom use (%/n)	ARR (%)	RRR%	With comprehensive interventions (%/n)	Without condom use (%/n)	ARR (%)	RRR%
2003	12,636	0.51 (65)	0.51 (65)	0.00	0.00	1.58 (200)	1.58 (200)	0.00	0.00	0.00 (0)	0.00 (0)	0.00	–
2004	16,479	0.56 (92)	0.56 (92)	0.00	0.00	1.77 (292)	1.77 (292)	0.00	0.00	0.00 (0)	0.00 (0)	0.00	–
2005	17,167	0.74 (127)	0.77 (133)	0.03	4.51	2.42 (415)	2.45 (421)	0.03	1.43	0.72 (3)	0.71 (3)	−0.01	−1.44
2006	18,476	0.91 (169)	1.02 (188)	0.10	10.11	3.12 (577)	3.26 (603)	0.14	4.31	1.03 (6)	0.99 (6)	−0.04	−4.46
2007	20,112	1.04 (210)	1.26 (253)	0.21	17.00	3.86 (777)	4.21 (846)	0.34	8.16	1.40 (11)	1.17 (10)	−0.23	−19.49
2008	22,624	1.11 (252)	1.45 (329)	0.34	23.40	4.50 (1017)	5.15 (1165)	0.65	12.70	1.07 (11)	0.85 (10)	−0.22	−25.73
2009	25,262	1.24 (314)	1.74 (439)	0.49	28.47	5.19 (1312)	6.29 (1588)	1.09	17.38	1.35 (18)	1.00 (16)	−0.36	−35.68
2010	26,669	1.29 (343)	1.90 (507)	0.61	32.35	6.09 (1624)	7.75 (2067)	1.66	21.43	1.81 (30)	1.34 (28)	−0.48	−35.71
2011	28,111	1.31 (369)	2.05 (577)	0.74	36.05	6.94 (1950)	9.25 (2601)	2.32	25.03	2.11 (42)	1.59 (42)	−0.52	−32.68
2012	29,589	1.27 (375)	2.13 (629)	0.86	40.38	7.67 (2268)	10.72 (3172)	3.06	28.50	2.37 (55)	1.80 (58)	−0.57	−31.85
2013	31,103	1.16 (360)	2.06 (642)	0.91	43.93	8.22 (2558)	12.01 (3736)	3.79	31.53	2.63 (69)	2.02 (77)	−0.61	−30.07

HIV incidence declined slightly with ART implementation among PLWHA of MSM in 2003–2013 in Chaoyang District. The ARR was from 0 to 0.04%, with an RRR% from 0 to 3.23%. HIV prevalence increased slightly, with ARR from − 0.29% to 0 and RRR% from 0 to 3.69%. HIV mortality decreased substantially. The ARR was from 0 to 1.75% and the RRR% was from 0 to 40.03% (as shown in Table 3, Figs. 1 and 2).

**Table 3:** Impact of ART on ARR and RRR% of HIV infections among MSM and HIV mortality among PLWHA of MSM in 2003–2013

		HIV incidence (per year)				HIV prevalence (per year)				HIV mortality (per year)			
Year	Num	With comprehensive interventions (%/n)	Without ART (%/n)	ARR (%)	RRR%	With comprehensive interventions (%/n)	Without ART (%/n)	ARR (%)	RRR%	With comprehensive interventions (%/n)	Without ART (%/n)	ARR	RRR%
2003	12,636	0.51 (65)	0.51 (65)	0.00	0.00	1.58 (200)	1.58 (200)	0.00	0.00	0.00 (0)	0.00 (0)	0.00	–
2004	16,479	0.56 (92)	0.56 (92)	0.00	0.00	1.77 (292)	1.77 (292)	0.00	0.00	0.00 (0)	0.00 (0)	0.00	–
2005	17,167	0.74 (127)	0.74 (127)	0.00	0.00	2.42 (415)	2.42 (415)	0.00	0.00	0.72 (3)	0.72 (3)	0.00	0.00
2006	18,476	0.91 (169)	0.91 (169)	0.00	0.00	3.12 (577)	3.12 (576)	−0.01	−0.17	1.03 (6)	1.20 (7)	0.17	14.29
2007	20,112	1.04 (210)	1.05 (211)	0.00	0.47	3.86 (777)	3.85 (774)	−0.01	− 0.39	1.40 (11)	1.65 (13)	0.26	15.49
2008	22,624	1.11 (252)	1.13 (256)	0.02	1.56	4.50 (1017)	4.46 (1008)	−0.04	−0.89	1.07 (11)	2.14 (22)	1.07	49.90
2009	25,262	1.24 (314)	1.27 (321)	0.03	2.18	5.19 (1312)	5.13 (1296)	−0.06	−1.23	1.35 (18)	2.41 (32)	1.06	43.83
2010	26,669	1.29 (343)	1.32 (351)	0.03	2.28	6.09 (1624)	6.00 (1599)	−0.09	−1.56	1.81 (30)	2.80 (46)	0.98	35.14
2011	28,111	1.31 (369)	1.34 (378)	0.03	2.38	6.94 (1950)	6.80 (1912)	−0.14	−1.99	2.11 (42)	3.29 (65)	1.18	35.87
2012	29,589	1.27 (375)	1.30 (385)	0.03	2.60	7.67 (2268)	7.47 (2209)	−0.20	−2.67	2.37 (55)	3.79 (87)	1.42	37.52
2013	31,103	1.16 (360)	1.20 (372)	0.04	3.23	8.22 (2558)	7.93 (2467)	−0.29	−3.69	2.63 (69)	4.38 (113)	1.75	40.03

HIV incidence declined substantially with a combination of ART and condom use among PLWHA of MSM in 2003–2013. The ARR was from 0 to 0.99% and the RRR% was from 0 to 46.11%. HIV incidence decreased slightly with ARR from 0 to 3.5% and RRR% from 0 to 29.88%. HIV mortality decreased substantially with ARR from − 0.01% to 1.02% and RRR% from-1.44% to 39.98% (as shown in Table 4, Figs. 1 and 2).

**Table 4:** Impact of condom use in combination with ART on ARR and RRR% of HIV infections among MSM and HIV mortality among PLWHA of MSM in 2003–2013

		HIV incidence (per year)				HIV prevalence (per year)				HIV mortality (per year)			
Year	Num	With comprehensive interventions (%/n)	Without Combined interventions (%/n)	ARR (%)	RRR%	With comprehensive interventions (%/n)	Without Combined interventions (%/n)	ARR (%)	RRR%	With comprehensive interventions (%/n)	Without Combined interventions (%/n)	ARR (%)	RRR%
2003	12,636	0.51 (65)	0.51 (65)	0.00	0.00	1.58 (200)	1.58 (200)	0.00	0.00	0.00 (0)	0.00 (0)	0.00	–
2004	16,479	0.56 (92)	0.56 (92)	0.00	0.00	1.77 (292)	1.77 (292)	0.00	0.00	0.00 (0)	0.00 (0)	0.00	–
2005	17,167	0.74 (127)	0.77 (133)	0.03	4.51	2.42 (415)	2.45 (421)	0.03	1.43	0.72 (3)	0.71 (3)	−0.01	−1.44
2006	18,476	0.91 (169)	1.02 (189)	0.11	10.58	3.12 (577)	3.26 (602)	0.14	4.15	1.03 (6)	1.15 (7)	0.12	10.46
2007	20,112	1.04 (210)	1.26 (254)	0.22	17.32	3.86 (777)	4.19 (843)	0.33	7.83	1.40 (11)	1.52 (13)	0.12	8.08
2008	22,624	1.11 (252)	1.48 (335)	0.37	24.78	4.50 (1017)	5.11 (1157)	0.62	12.10	1.07 (11)	1.78 (21)	0.71	39.98
2009	25,262	1.24 (314)	1.78 (450)	0.54	30.22	5.19 (1312)	6.23 (1575)	1.04	16.70	1.35 (18)	1.99 (32)	0.64	32.03
2010	26,669	1.29 (343)	1.95 (519)	0.66	33.91	6.09 (1624)	7.68 (2047)	1.59	20.66	1.81 (30)	2.24 (47)	0.43	19.19
2011	28,111	1.31 (369)	2.11 (593)	0.80	37.77	6.94 (1950)	9.14 (2569)	2.20	24.09	2.11 (42)	2.65 (70)	0.54	20.51
2012	29,589	1.27 (375)	2.20 (651)	0.93	42.40	7.67 (2268)	10.54 (3119)	2.88	27.28	2.37 (55)	3.11 (100)	0.74	23.79
2013	31,103	1.16 (360)	2.15 (668)	0.99	46.11	8.22 (2558)	11.73 (3648)	3.50	29.88	2.63 (69)	3.65 (138)	1.02	27.94

## Discussion

MSM maintain a relatively private status in China. It is challenging to diagnose HIV infections and subsequently carry out interventions aimed at reducing high-risk behaviour among MSM and implementing ART among PLWHA. We have used AEM to evaluate HIV preventive measures undertaken among MSM in 2003–2013 in Chaoyang, the district with the largest population in Beijing, based on community population statistics. Modelling data was obtained from community demography data of Chaoyang, routine monitoring data of HIV infection status among MSM, special investigations, data from voluntary consultation tests, and published references. This study should provide useful reference data for adjustments of future interventions to prevent the spread of HIV among MSM.

The MSM population has been recognized as the main source of new HIV infections in Chaoyang since 2001. Reducing HIV transmission among MSM can effectively control the progression of the HIV/AIDS epidemic in the district [32]. Condom use and ART were implemented among the MSM population from 2003 to 2013 in the Chaoyang District of Beijing. Compared with 2012, HIV incidence among MSM declined in 2013 and reached the target of the National AIDS Comprehensive Prevention and Control Demonstration Area (HIV incidence rates were reduced by 20%–25%) [33]. As shown in Table 2 to Table 4, we found that the decline of HIV incidence was estimated to have occurred without condom use and/or ART. One explanation could be that the exposure opportunities of the MSM population decreased due to increasing knowledge and the level of AIDS prevention in high-risk groups, including IDU, FSWs and multiple partners, in addition to the MSM population, along with the improvement of the AIDS prevention level in the whole community.

The rates of condom use between MSM and MSM during sex increased from 42.00% to 56.00% in 2003–2013. The rates of condom use also increased from 55.00% to 66.70% between MSM and MSW in 2003–2013. Our current data demonstrate that a simple increase in condom use among MSM resulted in a significant decrease in the relative risk of new HIV infections with an RRR% of 0 to 43.93%. Condom use also resulted in a decrease in the relative risk of current HIV infections from 0 to 31.53%. This finding is consistent with results from previous studies [34]. However, this study also found that the absolute risk of HIV-related death among PLWHA who are MSM increased slightly from 0 to 0.61% in 2003–2013 due to an increase in condom use. The relative risk of HIV-related death also increased slightly from 1.44% to 35.71%. Possible reasons for the increase in mortality were that the increase in condom use had no direct effect on HIV-related death among PLWHA with a large impact on reducing new HIV infections. It caused the number of new HIV infections to decrease, resulting in an annual reduction in the number of current HIV infections, thus leading to the increase in HIV mortality.

ART was first implemented in 2003 in Chaoyang. ART coverage increased from an initial 5.06% to 29.72% in 2013. ART coverage benefited from increased Voluntary Counselling and Testing (VCT) and regular follow-up visits to patients to promote ART treatment among those who qualified for the treatment standard. Our results suggest that HIV incidence among MSM was reduced with the implementation of ART in 2003–2013, which agrees with previous studies [35, 36]. In our study, we found that ART had limited effects in reducing new HIV infections, and the ARR percentage was from 0 to 0.04% and the RRR percentage was from 0 to 3.23%. It is possible that the effect of ART had not yet been given full attention because the coverage rate of ART was low in 2003–2013 in this area. Additionally, HIV mortality among MSM was dramatically decreased (RRR% from 0 to 40.03%), while both the HIV prevalence and the number of infection sources increased. We found that compared to condom use, the effect of ART on reducing new HIV infections was lower, while the effect of ART in reducing HIV mortality was greater, and HIV mortality clearly decreased after ART implementation.

Our study also found that the effect of condom use in combination with ART on reducing HIV incidence and HIV mortality was greater than the cumulative effect of either ART alone or condom use alone in 2003–2013. Based on the above results, condom use in combination with ART is more effective in preventing the spread of HIV among MSM in Chaoyang. Similar results were reported in the study [37] by Ramadanovic B, which suggested that expanding ART coverage combined with interventions targeting high-risk behaviours amplify the preventive impact.

We admit that this study has several limitations. The AEM is a semi-empirical model, so its effects on the estimation of HIV/AIDS epidemics and the assessment of interventions are restricted by the limitations of the design itself and the experience of the experts involved. In this study, HIV mortality was calculated by HIV deaths/PLWHA/year in AEM, and a more useful measure of the effect of the interventions on HIV mortality would be HIV deaths/100,000 population/year. In addition, this study does not take the influence of heterosexual behaviour of bisexual people among MSM into consideration. The number of heterosexual partners such as FSW and changes in their high-risk behaviour may impact HIV infections among MSM. Moreover, we did not consider drug resistance and ART failure, which may theoretically influence the efficacy of ART.

## Conclusions

In conclusion, interventions aimed at preventing HIV spread among MSM were effective in the Chaoyang District of Beijing in 2003–2013. Condom use can reduce the absolute and relative risk of HIV incidence and HIV prevalence. ART decreased the absolute risk and relative risk percentage of HIV mortality among PLWHA of MSM. Among MSM, condom use in combination with ART not only reduces HIV incidence but also reduces HIV mortality, and it is more effective in the prevention and control of the spread of HIV.

## Abbreviations

AEM: Asian Epidemic Model
ARR: Absolute risk reduction
ART: Antiretroviral therapy
FSWs: Female sex workers
IDU: IV drug user
MSM: Men who had sex with men
MSW: Male sex worker
PLWHA: People living with HIV/AIDS
RRR: Relative risk reduction
USAID: United States Agency for International Development
